# Passive tracking of underwater acoustic targets based on multi-beam LOFAR and deep learning

**DOI:** 10.1371/journal.pone.0273898

**Published:** 2022-12-01

**Authors:** Maofa Wang, Baochun Qiu, Zefei Zhu, Li Ma, Chuanping Zhou

**Affiliations:** 1 School of Mechanical Engineering, Hangzhou Dianzi University, Hangzhou, China; 2 Key Laboratory of Underwater Acoustic Environment Institute of Acoustic, Chinese Academy of Science, Beijing, China; 3 College of Electrical Engineering, Zhejiang University; Hangzhou, China; University of Bonab, ISLAMIC REPUBLIC OF IRAN

## Abstract

Conventional passive tracking methods for underwater acoustic targets in sonar engineering generate time azimuth histogram and use it as a basis for target azimuth and tracking. Passive underwater acoustic targets only have azimuth information on the time azimuth histogram, which is easy to be lost and disturbed by ocean noise. To improve the accuracy of passive tracking, we propose to adopt the processed multi-beam Low Frequency Analysis and Recording (LOFAR) as the dataset for passive tracking. In this paper, an improved LeNet-5 convolutional neural network model (CNN) model is used to identify targets, and a passive tracking method for underwater acoustic targets based on multi-beam LOFAR and deep learning is proposed, combined with Extended Kalman Filter (EKF) to improve the tracking accuracy. The performance of the method under realistic conditions is evaluated through simulation analysis and validation using data obtained from marine experiments.

## Introduction

Sonar systems are widely used for underwater detection, identification, localization, navigation and communication, and are usually divided into active and passive sonar systems according to their mode of operation [1]. Unlike active sonar, passive sonar itself does not emit any signal, but only relies on the received acoustic signals to analyze the ocean environment situation, detect potential targets and start the corresponding signal processing. Passive sonar is well suited for hydroacoustic target detection and tracking activities that require a high degree of concealment.

The existing underwater acoustic target passive tracking system obtains the time azimuth histogram to detect the target and track the target through beam forming, which has low information utilization rate for the sonar array, and the obtained azimuth belongs to nonlinear information [2]. Since there is only the azimuth information of the target on the time azimuth histogram, it may occur that the azimuth trajectories of two or more targets cross, which may easily lead to the loss of the target azimuth trajectory [3]. The weak target signal and the interference of ocean noise all increase the difficulty of passive tracking.

The data association between the observed data and the already existing azimuth trajectory is the key problem to be solved for passive tracking of underwater acoustic targets, which is related to the ability to correctly distinguish different targets and track them [4]. Various algorithms have been proposed for hydroacoustic multi-target tracking. The nearest neighbor method based on decision-making has the advantages of small amount of calculation and simple implementation, but it cannot correctly associate the two close targets [5]. Methods based on probabilistic statistics are multiple hypothesis correlation (MHT) [6] and joint probabilistic data association (JPDA) [7], which can have an impact on the calculation of their weights when there is relatively strong clutter in the measurements, making it impossible to assign the measurements correctly. The fuzzy logic-based association methods are fuzzy data association and fuzzy clustering association. Fuzzy clustering association divides the known data into several classes, which minimizes the objective function of non-similarity metrics [8], and the assignment of measurements and waypoints may still be wrong in a dense clutter environment. Therefore, it is necessary to study how to obtain more information of underwater acoustic targets for tracking in the field of passive tracking of underwater acoustic targets. The methods based on separated features are spectral feature correlation, sub band peak correlation [9]. The separation feature-based algorithm requires manual extraction of the target signal and then reduces it to spectral line features, which has the shortcomings of incomplete feature extraction and huge computational effort.

Deep learning is a machine learning theory and method that uses nonlinear information processing techniques to achieve multilevel, supervised or unsupervised feature extraction and transformation, and to perform pattern analysis and classification [10]. Deep learning has excellent performance in the field of speech processing, optimal decision making, and many scholars are currently studying the application of deep learning techniques in hydroacoustic engineering [11]. Researchers have proposed to use neural network models such as long and short term memory networks (LSTM), CNN, and deep confidence networks (DBN) [12] in deep learning to obtain feature information of targets and build recognition models with good results. Ding [13] proposed a supervised method based on deep neural networks (DNNs) to jointly estimate the azimuth and distance of binaural signals, and achieved good results. Nie [14] proposed a reconstruction-based DNN method to process incomplete target categories in the training set, and verified the feasibility under different signal-to-noise ratios. Yang [15] proposed a multi-attribute correlation perception method for underwater acoustic targets based on deep learning, which uses the correlation between the multi-attributes of underwater acoustic targets for modeling and training, and its recognition accuracy has been improved. There have been many studies on the application of neural network to pure underwater acoustic target recognition and classification, but there are not many studies on passive tracking of underwater acoustic targets in actual sonar engineering.

In our previous research on the active tracking method of underwater acoustic targets based on deep learning, we mainly carried out matched filtering for signals between specific frequency bandwidths, and identifies targets and reverberation according to the bright spot features of the targets, which are used to confirm the correlation between targets and tracks [16]. Different from active tracking, the frequency spectrum of passive tracking target is not fixed in a specific frequency band, and there is no bright spot feature model to identify, and it is easily masked by ocean noise. During the tracking process, different target signals in the data received by the sonar are mixed together, and it is necessary to distinguish different target signals and determine the target azimuth. The key point of identification in passive tracking is how to obtain and distinguish the self-radiated noise characteristics of underwater acoustic targets.

LOFAR and Noise envelope demodulation spectrum (DEMOM) are often used to identify underwater acoustic targets. LOFAR analysis can extract the line spectrum by direct FFT. In DEMON analysis, the envelope of the signal must be demodulated first, and then the FFT analysis is performed, which increases the complexity of the process of identifying and tracking the target. In this paper, we propose to adopt the multi-beam LOFAR spectrogram as the input layer. The multi-beam LOFAR spectrogram contains the time and space information of the target, which makes the multi-dimensional characteristics of the target more prominent and effectively reduces the interference of noise and other target signals.

In this paper, we propose a passive tracking method for underwater acoustic targets based on multi-beam LOFAR and deep learning combined with EKF (MLDE) to improve the tracking accuracy. We improved the LeNet-5 model [17] for passive object recognition, and adjusted the size of the input layer according to multi-beam LOFAR. Due to the small amount of data of underwater acoustic target samples, it is easy to overfit in the training process, we use the dropout mechanism to improve the generalization of the model. We use the ShipsEar database [18] to verify the recognition accuracy of the model, which can accurately identify different tracked targets. To further improve the tracking accuracy, EKF is used in the passive tracking process. Finally, we use the simulation data and actual sea trial data to verify the passive tracking method of underwater acoustic targets.

The rest of the paper is as follows: The second part introduces the composition and method of our framework, including data preprocessing, construction of CNN model, and training and validation; the third part is the target passive tracking process; the fourth part is the simulation analysis, and the fifth part is the sea trial data analysis and verification; and the sixth part is the conclusion.

## Our framework

### Structure

**Fig 1:**
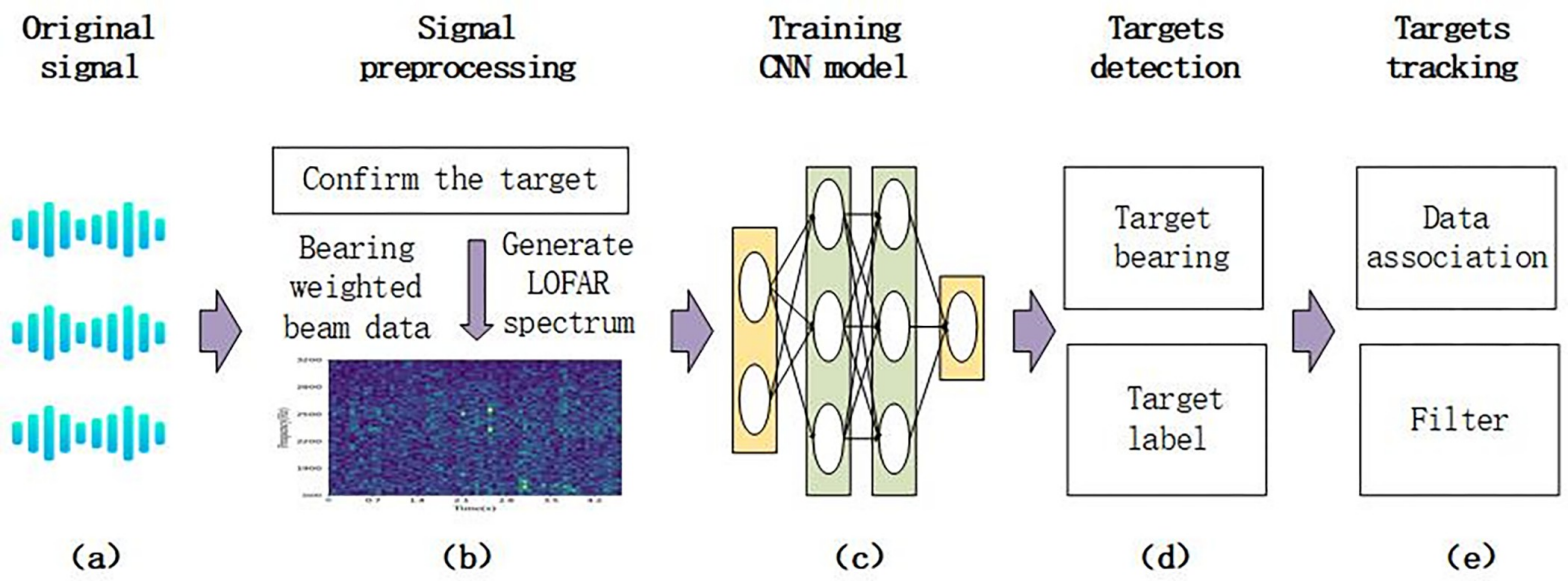
Framework and process flow in the MLDE method. (a) shows the raw sonar array signal. (b) is the pre-processing of the data after confirming the target, azimuth weighting, and generating the multi-beam LOFAR spectrum, which can be used for CNN model training. (c) is the training of the CNN model. (d) The trained CNN model is used to identify the target and give the azimuth measurements. (e) The azimuth measurements and the trajectory are correlated and filtered by EKF.


Fig 1 shows the overall structure of the MLDE method framework, and the whole process can be represented by Formula (1):

$$\tilde{y}=\mathrm{Pre}(\omega ⊗S),\;\mathrm{then}\;F(L,\theta )=P(cnn(\tilde{y}))$$ (1)

In Eq (1), *S* is the original sonar array signal, and $\tilde{y}$ is the image data obtained that can be used for CNN model training. Pre(⋅) indicates that after azimuthal weighting of the sonar array signal, the weighted data are converted into multi-beam LOFAR spectrum by frequency domain–time domain data conversion. Since the multi-beam LOFAR spectrum generation process goes through color gamut mapping, the energy spectrum values are converted to gray or RGB color values, which can be applied to the next step of CNN model training and recognition, denoted by *cnn*(⋅). *P*(⋅) denotes the CNN model trained to recognize the data to be recognized after azimuth weighting to obtain *F*(*L*,*θ*) of the target, including the classification label *L* and azimuth *θ* of the target. In the final data association, combined with the EKF, the recognition results of the CNN model are further revised to complete a passive tracking process.

### Data pre-processing

In order to train the model, the target signal needs to be extracted from the sonar array signal containing clutter to generate the dataset.

**Fig 2:**
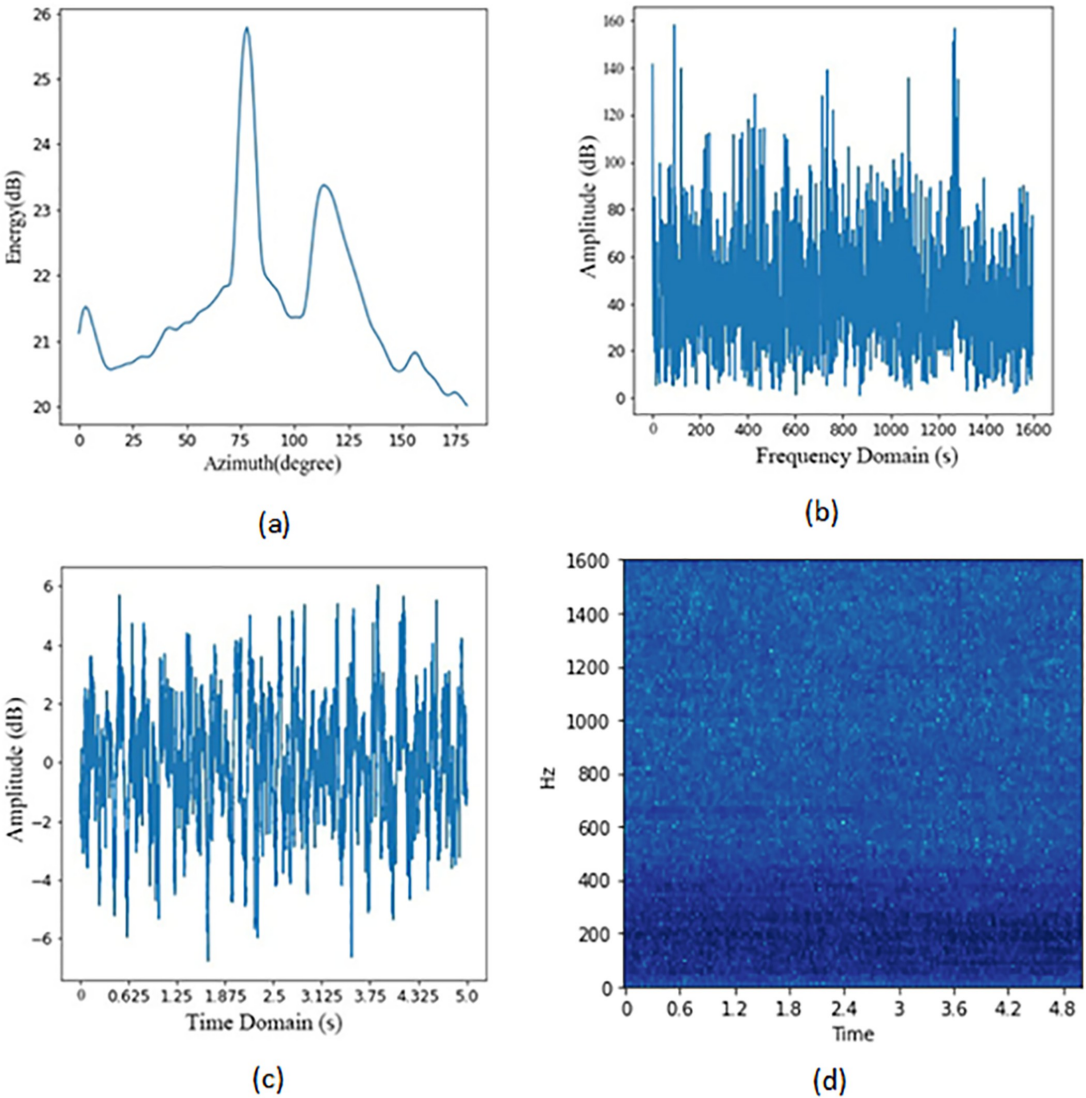
Target multi-beam LOFAR spectrogram obtained in a single collection. (a) Spectrogram of spatial energy distribution obtained by beamforming, (b) The frequency domain data waveform after target bearing weighting, (c) Time-domain data waveform after target azimuth weighting, (d) Multi-beam LOFAR spectrogram of the target.

The sample collection process for a single target is shown in Fig 2. The first step uses beamforming to process the array signal to obtain the spatial azimuth energy distribution map, and the maximum value method is used to determine the existence of the target. The second step is to weight the array signal according to the azimuth information to obtain the frequency domain signal of the beam where the target is located. The third step is to convert the frequency domain into time domain data. The fourth step is to convert the time domain data into multi-beam LOFAR spectrograms using short-time Fourier transform.

The purpose of the first step is to process the purest possible target signal from the sonar array. Beamforming is a process of converting the sonar array signal from time domain information to spatial domain information.

For a uniform line array containing *M* elements with an interval of *d*, the received sonar array signal is first Fourier transformed to obtain the array frequency domain data, as shown in Formula (2).


$$X_{i}(f)=\int_{-\infty }^{+\infty }s_{i}(t)e^{-j\omega t}dt\;t=1,2,\ldots ,T$$ (2)

In Formula (2), *t* is the sampling moment of the signal, and then the weighting vector *W*(*θ*,*f*) is calculated.


$$W(\theta ,f)=e^{j2\pi f(i-1)d\cos\theta /c}$$ (3)

In Formula (3), *i* is the serial number of array elements in the array; *d* is the array element interval, unit is *m*; *c* denotes the speed of sound, the unit is *m/s*; *f* is the frequency. The unit is *Hz*. The array frequency domain data are multiplied with the weighted vectors to obtain the frequency domain data for different beams. The array frequency domain data are multiplied with the weighted vector to obtain the beam frequency domain data for different beam pointing angles.


$$Y(\theta ,f)=\sum_{i=1}^{M}W_{i}^{H}(\theta ,f)X_{i}(f)$$ (4)

In Formula (4), $W_{i}^{H}(\theta ,f)$ is the covariance transpose matrix of the weighted vector. The spatial spectrum is obtained by summing the squared frequency domain data of all beams, as shown in Fig 2(A).

In Fig 2(A), the x-axis is the azimuth and the y-axis is the energy intensity, the unit is *dB*. After obtaining the spatial azimuth energy distribution map, the energy threshold is determined according to the actual marine environmental noise, and the potential target is determined in combination with the maximum value method.

In the second step, according to the azimuth of the target, the array signal is weighted to obtain the beam frequency domain data corresponding to the target. As show in Fig 2(B), the x-axis is the frequency and the y-axis is the amplitude. The information of the target is analyzed continuously on the time axis, and the frequency domain signal is converted to the time domain signal as a way to obtain the time domain accumulation of the signal. This is shown in Fig 2(C).

The multi-beam LOFAR spectrum is obtained by processing the time domain data using the short-time Fourier transform, and the steps include framing, adding windows, Fourier transform, finding the power spectrum, color gamut mapping and superposition [19]. The multi-beam LOFAR spectrum contains both frequency and time domain information of the signal, demonstrating the change of energy distribution in the target frequency domain with time. Through a series of transformations, the information of the target is extracted and converted into a form convenient for deep learning, which is the antecedent process to generate the target dataset. The target time-domain data are converted to LOFAR spectrum as shown in Fig 2(D).

When generating a LOFAR spectrum, the energy spectrum is converted to grayscale or RGB color values by color gamut mapping. The LOFAR spectrum is stored as a two-dimensional array, and the target label information is added to generate a multi-dimensional array to deposit the data. These steps are repeated to obtain samples with different objectives to deposit in the dataset.

### CNN model

CNN has been proposed in the 1980s. In 1998, Lecun applied the Back Propagation (BP) algorithm to the training of neural network structures and proposed LeNet for handwritten digit recognition [17]. 2021, the Alexnet model [20], introduced a new deep structure and dropout method, which greatly improved the correct rate of image recognition.

The typical structure of a CNN contains convolutional layers, pooling layers, and fully connected layers [21], and the convolutional and pooling layers usually contain multiple feature images. The data can be transformed from a two-dimensional matrix to a one-dimensional feature vector by multi-layer convolution and pooling. Finally, the predicted category labels can be obtained through classification layers such as Softmax.

**Fig 3:**
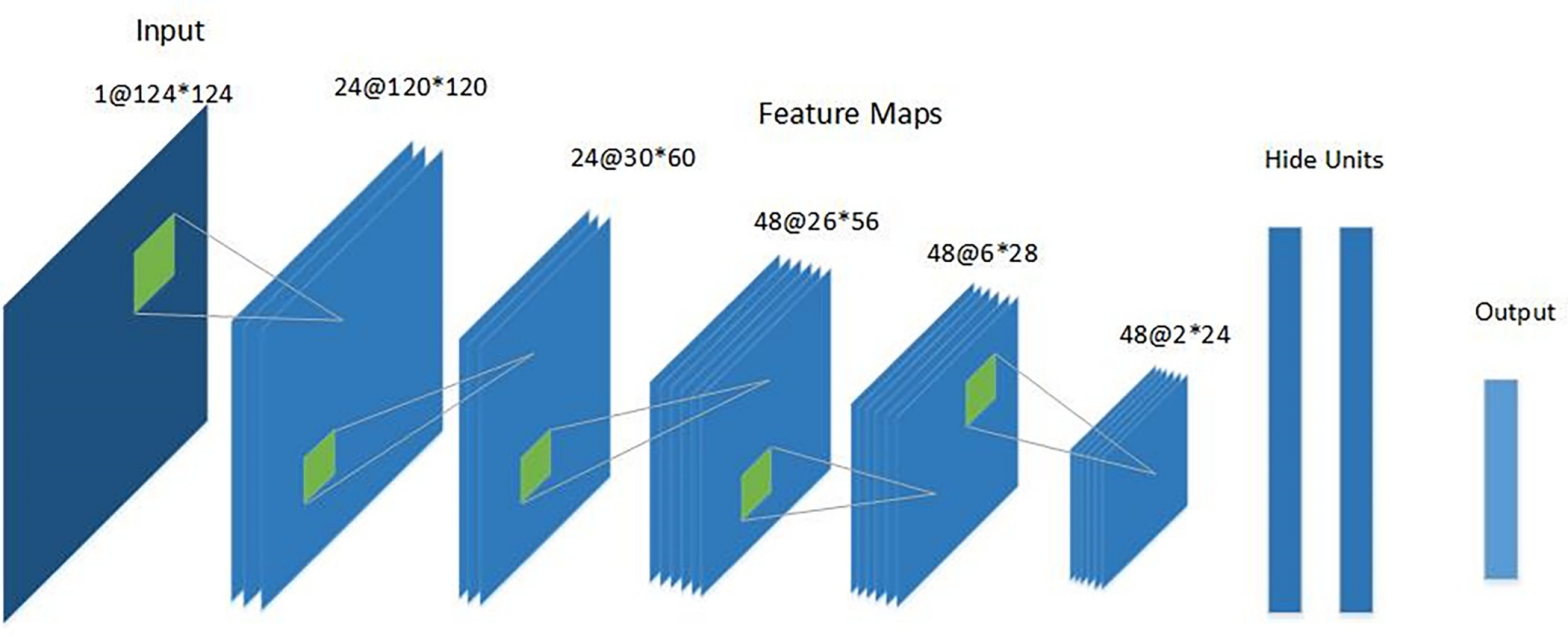
CNN model structure schematic diagram.

We improved the convolutional neural network model based on LeNet-5 and added a dropout mechanism to improve the generalization of the model. The CNN model proposed in this paper consists of three convolutional layers, two maximum pooling layers and two fully connected layers, and the dropout method is used for training, and the activation function is a Softmax function. Fig 3 shows the schematic diagram of the CNN model used in this paper.

A number of feature planes consisting of rectangularly arranged neurons contained in the convolutional layer of the model are multiplied with the corresponding positions using a convolutional kernel. By convolution operation, the original signal features are enhanced and noise is reduced. The output of the convolution layer is,

$$s(u,v)=\sum_{m}\sum_{n}x(i+m,j+n)w(m,n)+b$$ (5)

The pooling layer, also known as downsampling, is mainly used to down sample the features of the hydroacoustic target, compress the number of data and parameters, reduce overfitting, and improve the fault tolerance of the model. Both pooling layers of the model use maximum pooling, selecting the maximum value in the window as the sampling value and retaining the strongest feature input in the region.

If the neural network model has too many parameters and too few training samples, the trained model will easily produce overfitting. AlexNet solves this problem using the dropout method to improve the performance of the neural network by blocking the joint action of feature detectors. During training, a certain neuron stops working with a certain probability, which makes the model more generalizable because it does not rely too much on certain local features. The model in this paper uses the dropout method at the flatten layer as well as at the first connection layer, which stops working with a 50% probability at each training. The last fully connected layer in the model uses Softmax to act as a classifier. The output {*y*_1_,*y*_2_,…*y*_*n*_} from each node of the previous neural network layer is used as the confidence level to generate the new output $e^{y_{i}}$. Formula (6) gives the method for calculating the probability of target attribution categories using Softmax.


$$p_{i}=Softmax(y_{i})=(\frac{e^{y_{i}}}{\sum_{i=1}^{n}e^{y_{i}}})$$ (6)

Compared with the deep neural network model we used in active tracking before, the CNN model used in this paper is simpler and adopts the dropout mechanism. This is considering that in the actual sonar project, due to the small number of target samples, the use of deep neural systems can easily lead to overfitting, and when multiple convolutional layers are applied, the node features of the target tend to converge to the same vector and gradually become indistinguishable [22].

**Fig 4:**
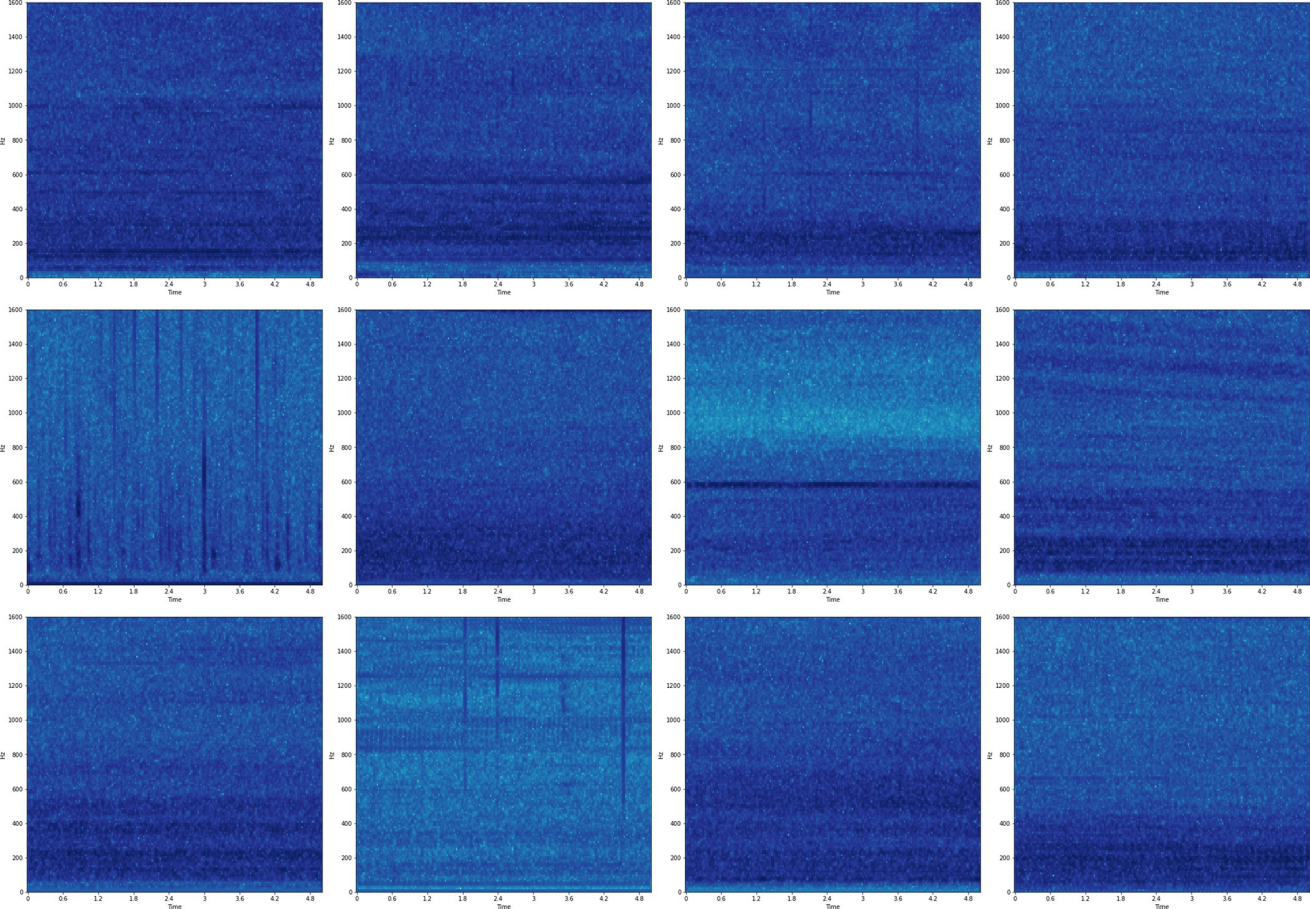
LOFAR spectrum of different samples. Convert raw sound data in ShipsEar database to LOFAR spectrogram.

We validated the model recognition accuracy using the ShipsEar database. ShipsEar is a database of underwater marine sounds aimed at providing researchers with sound recordings of vessels of various types and natural background noise, including fishing boats, ocean liners, ferries of various sizes, container, roro, tugs, pilot boats, yachts, small sail boats [18]. Fig 4 shows the LOFAR spectrum of the sample obtained after processing in the ShipsEar database.

**Fig 5:**
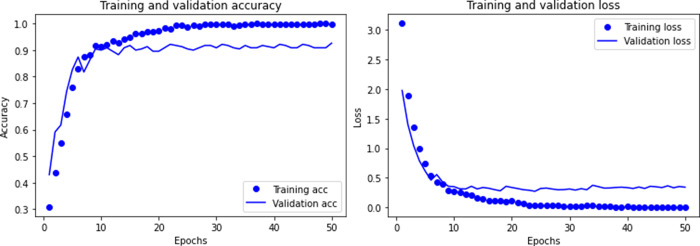
Accuracy and loss of CNN model when training 50 times.

**Fig 6:**
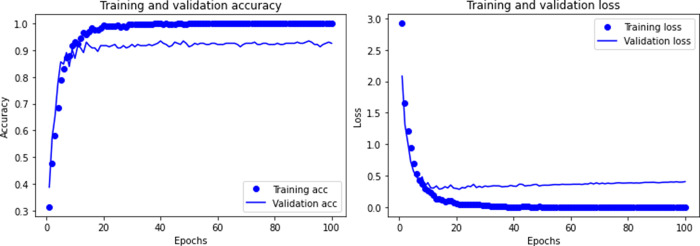
Accuracy and loss of CNN model when training 100 times.

**Fig 7:**
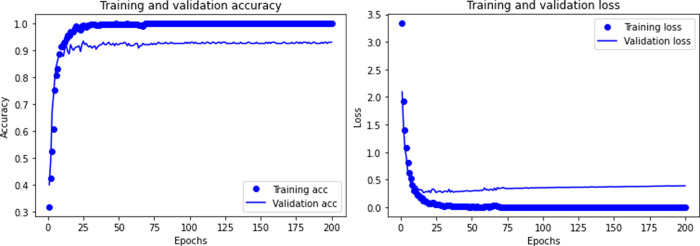
Accuracy and loss of CNN model when training 200 times.

The sample contains 11 types of ship data and marine environmental noise. After removing invalid data, there are a total of 920 samples. Then we trained the CNN model, and Figs 5–7 shows the recognition accuracy of the model after different times of training.

Comparing with different training times, the recognition accuracy of CNN model increases with the increase of training times, but this improvement is not obvious. Due to the small sample data of underwater acoustic targets, too many training times lead to over-fitting, and the recognition accuracy declines.

Compared with the underwater acoustic target classification method proposed by Yin [23] based on the deep sound feature extraction network of VGGNet, the recognition accuracy is between 94% and 98%, our proposed model is slightly insufficient in accuracy. This may be due to the relative simplicity of our model, the uneven number of samples in the training data, and the addition of environmental noise. Through training tests, the model designed in this paper can identify hydroacoustic targets very accurately and output the class labels of the targets. Since the input data to be detected is a multi-beam LOFAR spectrogram containing azimuth information, the target type and target azimuth identified in this detection can be obtained from the recognition results of the model.

### Extended Kalman filtering algorithm

The predictions given by deep learning models are not always reliable. In areas with high safety requirements such as autonomous driving, relying entirely on deep models for decision-making may lead to catastrophic consequences [24]. When a new target is encountered during tracking, the model may force a prediction. Therefore, we need to add a filter to ensure that the prediction results are in a correct range.

In this paper we use EKF to further improve the accuracy of passive tracking. EKF is a nonlinear filtering algorithm, which is suitable for passive tracking in nonlinear systems with only azimuth information, and can achieve good results [25].

As the passive tracking of hydroacoustic targets belongs to pure azimuth tracking, the measured target azimuth information is used to estimate the motion parameters of the target, so as to achieve continuous tracking of the target, which is a nonlinear system. In this paper, the discrete nonlinear system state and observation equations are used to construct the motion model of the target, as shown in Formula (7) and Formula (8).


X(k)=ΦX(k−1)+ΓU(k) (7)

$$Z(k)=\arctan(\frac{y(k)-y_{0}}{x(k)-x_{0}})+V(k)$$ (8)

In Formulae (7) and (8), *X*(*k*) is the state at moment *k*, *Z*(*k*) is the observed signal of the corresponding state, Φ is the system state function, Γ is the noise driving matrix, *U*(*k*) is the process noise, and *V*(*k*) is the observed noise.

The extended Kalman filter is a simple and effective estimation algorithm for nonlinear systems that can approximate the linearization of the nonlinear system and then apply the Kalman filter to complete the filtered estimation of the target [26]. The algorithm steps are as follows:

$$\hat{X}(k+1|k)=f[k,\hat{X}(k|k)]$$ (9)

Formula (9) is used to calculate the predicted value of the state, *f*[⋅] is a nonlinear state function, $\hat{X}(k|k)$ is the state value at time *k*, $\hat{X}(k+1|k)$ is the predicted value at time *k+1*.


$$P(k+1|k)=\Phi (k+1|k)P(k|k)\Phi^{T}(k+1|k)+Q$$ (10)

Formula (10) is used to solve for the predicted value of the state covariance. **Φ**(*k*+1|*k*) is the state transition matrix, **P**(*k*|*k*) is the state covariance matrix. **P**(*k*+1|*k*) is the predicted value of the covariance matrix.


$$K=P(k+1|k)H^{T}(k+1){\left[H(k+1)P(k+1|k)H^{T}(k+1)+R\right)}^{-1}$$ (11)

Formula (11) is used to solve for the Kalman gain **K**. **H**(*k*+1) is the observation matrix, **R** is the variance of the measurement noise.


$$\hat{X}(k+1|k+1)=\hat{X}(k+1|k)+K\{Z(k+1)-h[k,\hat{X}(k+1|k)]\}$$ (12)

Formula (12) is used to solve for the estimated state $\hat{X}(k+1|k+1)$ after target filtering, and *h*[⋅] is the observation function.


P(k+1)=[I−KH(k+1)]P(k+1|k) (13)

Formula (13) is used to update the covariance **P**(*k*+1), **I** is the unit matrix.

In contrast to the basic equations of the linear Kalman filter, the state transfer matrix and observation matrix can be replaced by Jacobi matrices of the state transfer function and the observation function.


$$\begin{array}{l} H=\frac{\partial Z(k)}{\partial X(k)}=\left[\frac{\partial Z(k)}{\partial x(k)},\frac{\partial Z(k)}{\partial \dot{x}(k)},\frac{\partial Z(k)}{\partial y(k)},\frac{\partial Z(k)}{\partial \dot{y}(k)}\right]\\ =\left[\frac{-(y(k)-y_{0})}{{\left(x(k)-x_{0}\right)}^{2}+{\left(y(k)-y_{0}\right)}^{2}},0,\frac{\left(x(k)-x_{0}\right)}{{\left(x(k)-x_{0}\right)}^{2}+{\left(y(k)-y_{0}\right)}^{2}},0\right] \end{array}$$ (14)

Formula (14) is the Jacobian matrix of the observation equation. (*x*(*k*),*y*(*k*)) is the position of the tracked target, $\left(\dot{x}(k),\dot{y}(k)\right)$ is the speed of the tracked target, (*x*_0_,*y*_0_) is the location of the observatory.

Formula (7) to Formula (14) are linked and can be used for passive target tracking. Each target azimuth observation is given by CNN model, which is combined with extended Kalman filtering to achieve filtering and estimation of the target state.

## Simulation verification

### Simulation signal setting

**Fig 8:**
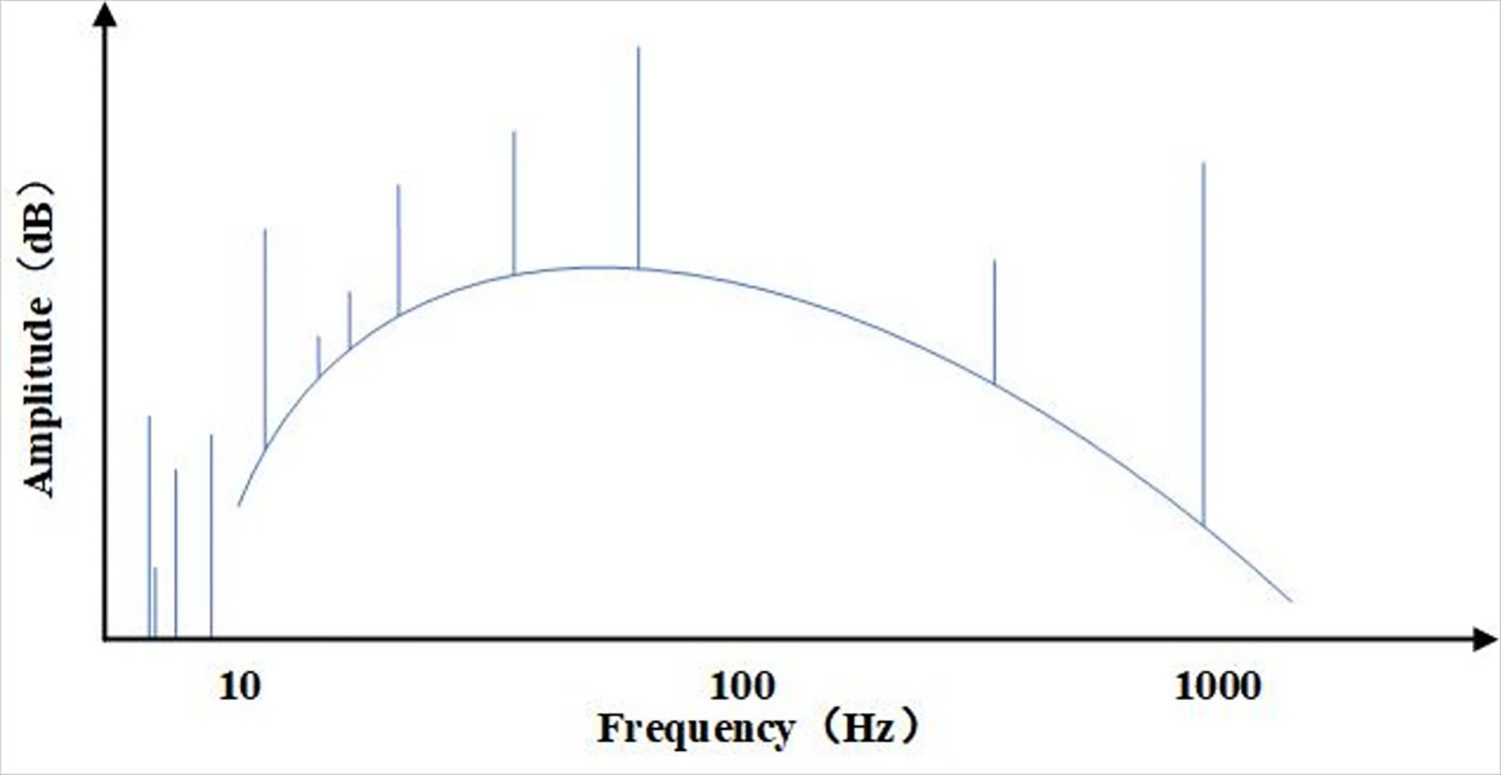
Schematic diagram of the composition of the ship’s radiated noise spectrum.

In order to verify the performance of the MLDE method, we use computer simulations of the data received by the passive sonar array for validation. Many scholars have conducted a lot of research on typical naval radiated noise in hydroacoustic targets, which typically consists of a broadband continuous spectrum and a series of line spectra [25], as shown in Fig 8.

The broadband continuous spectrum is composed of continuous frequency components, and the set of equations shown in Formula (14) is its generation process.


$$\begin{array}{l} X(k)=fft[x(m)]\\ P(k)=H(k)X(k)\\ P(m)=ifft[P(k)] \end{array}\}$$ (15)

In Formula (15), *x*(*m*) is a Gaussian white noise random sequence, which is Fourier transformed to obtain its frequency domain sequence *X*(*k*), *H*(*k*) is the specific frequency response filter and *P*(*k*) is the filtered frequency domain sequence. Finally, after the inverse Fourier transform, the broadband continuous spectrum time domain sequence *P*(*m*) is obtained to meet the requirements. The ship radiated noise line spectrum components are mainly related to its power system and propulsion and are considered to be composed of a series of harmonic components [27], can be expressed as,

f=nms (16)

In Formula (16), *s* is the propeller speed in r/min, *n* is the number of propeller blades, and *m* is the number of harmonics.

### Simulation study

**Fig 9:**
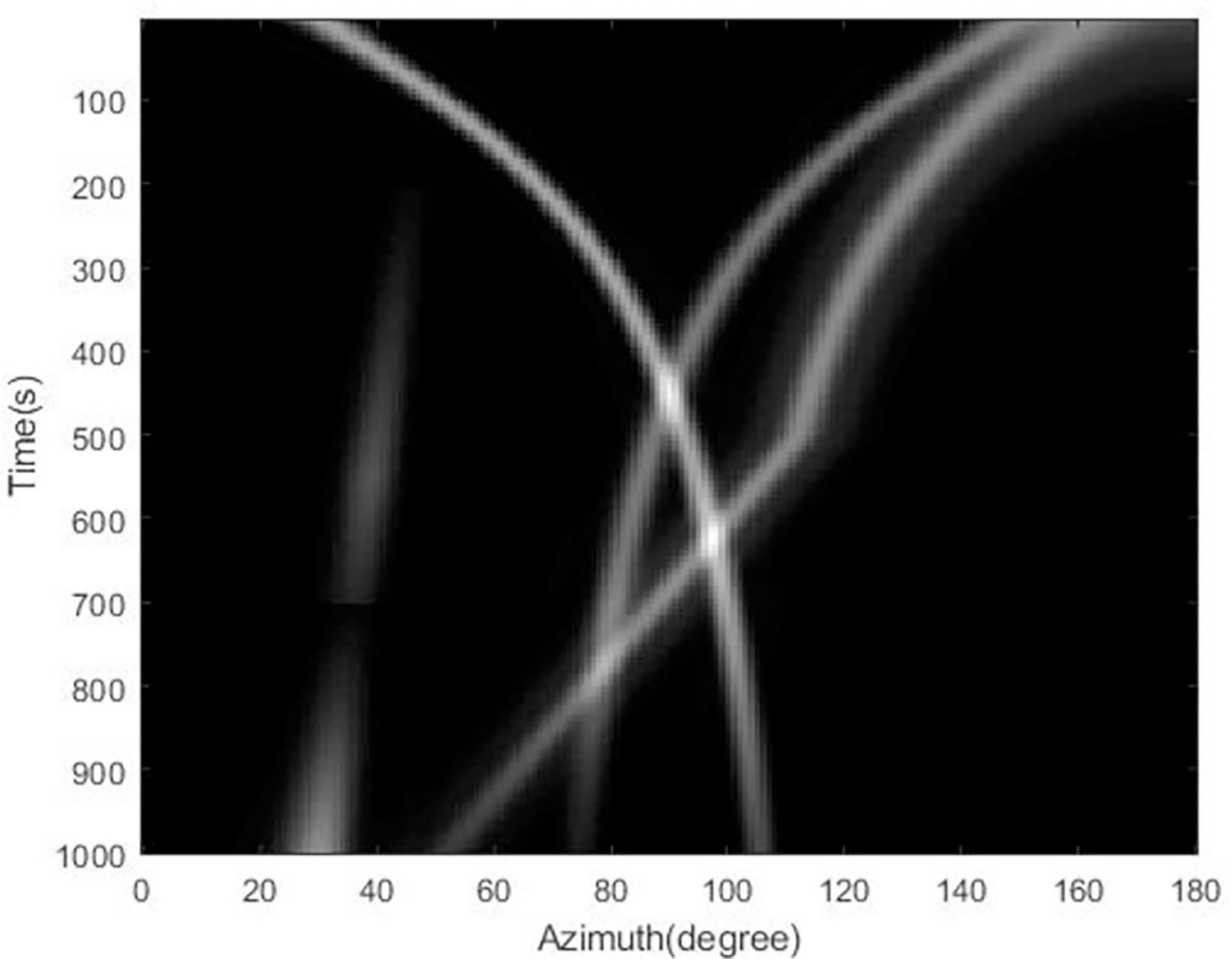
Time azimuth histogram obtained from processing simulation data.

In the simulation, the number of array elements of the passive sonar array is 32, the distance between the array elements is 6m, and four moving targets are set. Using the beam formation mentioned in data pre-processing, the spatial spectrum can be obtained, and after time accumulation, the azimuth-time history chart is obtained, as shown in Fig 9. Multiple moving targets with overlapping, interrupted and disappearing azimuth trajectories can be seen in the figure.

**Fig 10:**
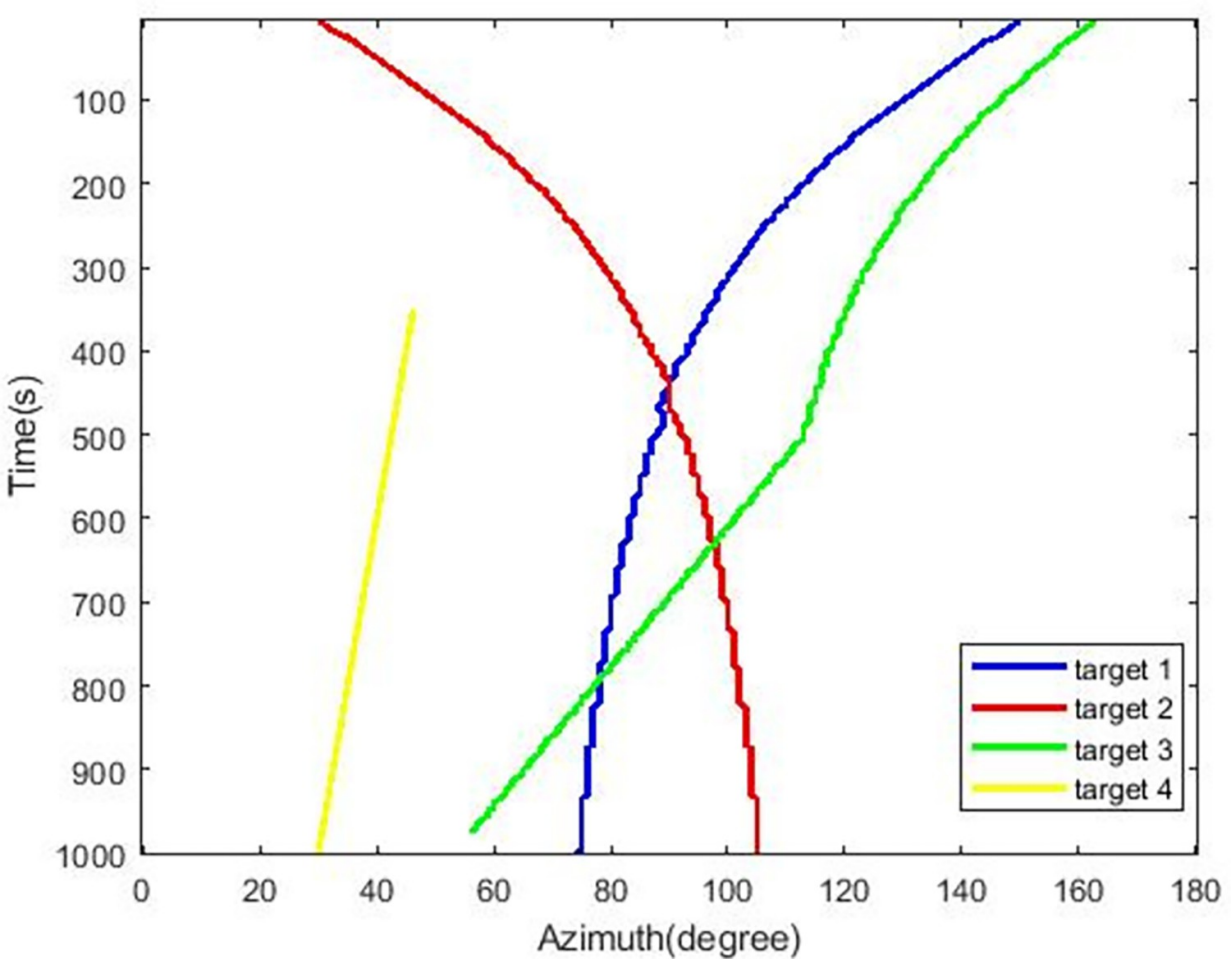
Simulated target azimuth over time.

The simulated target azimuths over time are shown in Fig 10.

**Fig 11:**
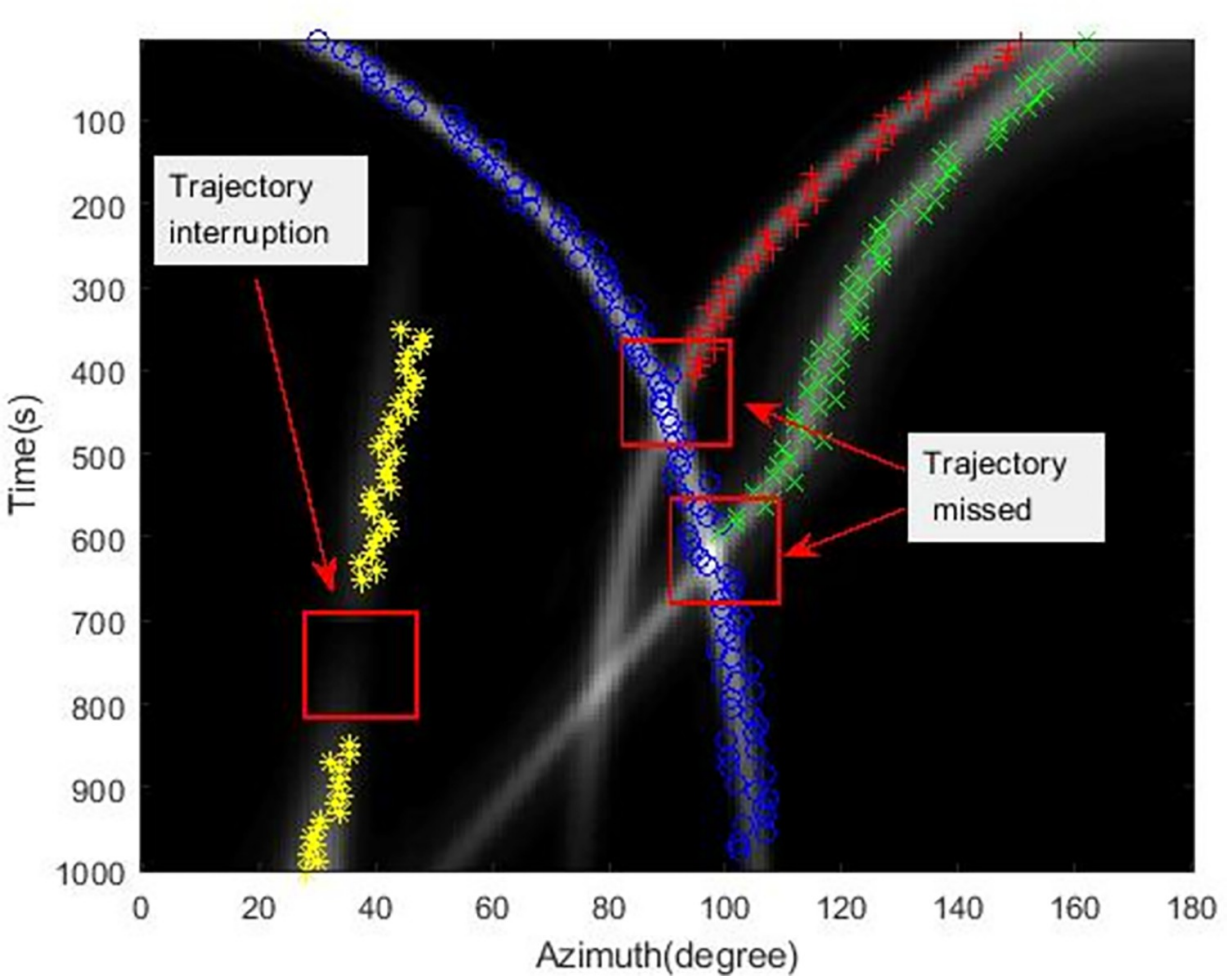
Simulation tracking results of the EKF-Max method.

**Fig 12:**
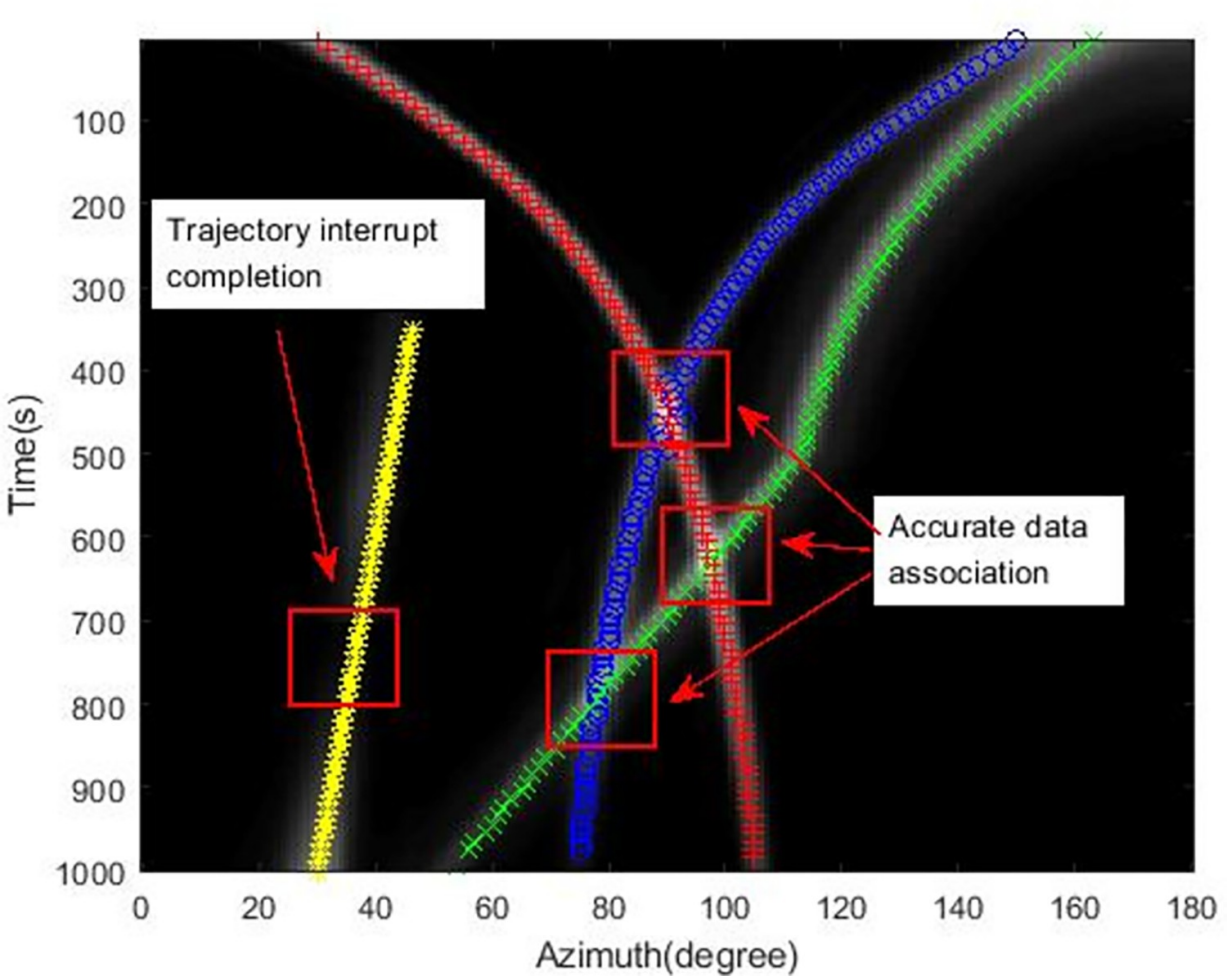
Simulation tracking results of the MLDE method.

The tracking results using the extended Kalman filter with Max value (EKF-Max) method and the MLDE method are shown in Figs 11 and 12.

It can be seen from Fig 11 that with the EKF-Max method, the azimuth trajectory of one target is lost when the target azimuth trajectory is crossed. And the azimuth trajectory is interrupted because the target signal is weak and the target cannot be detected.

As can be seen from Fig 12, the MLDE method can accurately associate the data and ensure the continuity of the bearing trajectory after the crossover of different targets occurs. When the target signal is weak, the target is still detected and a complete azimuth trajectory is generated.

To further quantitatively estimate the performance of the method proposed in this paper, the tracking bias of the two methods is compared, and the following formula is used here to measure the tracking bias.


$$\mathrm{RMS}=\frac{1}{n}\sum_{i=1}^{n}\left|x{\left(i\right)}^{*}-x(i)\right|$$ (17)

In Formula (17), *x*(*i*)* is the measured azimuth value, *x*(*i*) is the azimuth value in the preset motion azimuth trajectory, and *n* is the number of azimuth measurements. The tracking bias and average bias for each target are listed in Table 1.

**Table 1:** Comparison of tracking azimuth deviation.

	Target 1	Target 2	Target 3	Target 4	Mean error
**EFK-Max**	2.215°	2.195°	2.203°	1.595°	2.015°
**EKF-DL**	1.210°	1.185°	1.195°	0.675°	1.066°

From the simulation results, the EKF-DL method can accurately correlate different targets when there are cross-targets when tracking multiple targets, and the tracking bias is smaller compared to the EFK-Max method.

## Sea trial data verification

### Sea trial data details and analysis

In order to verify the effectiveness of the MLDE method in practical applications, we have used actual marine experimental data to validate it. The data were collected from a passive sonar array with a length of 186 m and an array element spacing of 6 m, containing 32 hydrophones.

The sea trial data comes from a sea trial in a certain area of the South China Sea in 2019. The working performance of passive sonar was tested in the experiment. This is a piece of sonar data that contains two passive underwater acoustic targets, along with some transmitted pulses. Due to the lack of latitude and longitude changes of passive underwater acoustic targets, we first analyzed the data before using the MLDE method to process the data, mainly to determine the azimuth changes of the passive targets for comparison with the results of the MLDE method.

**Fig 13:**
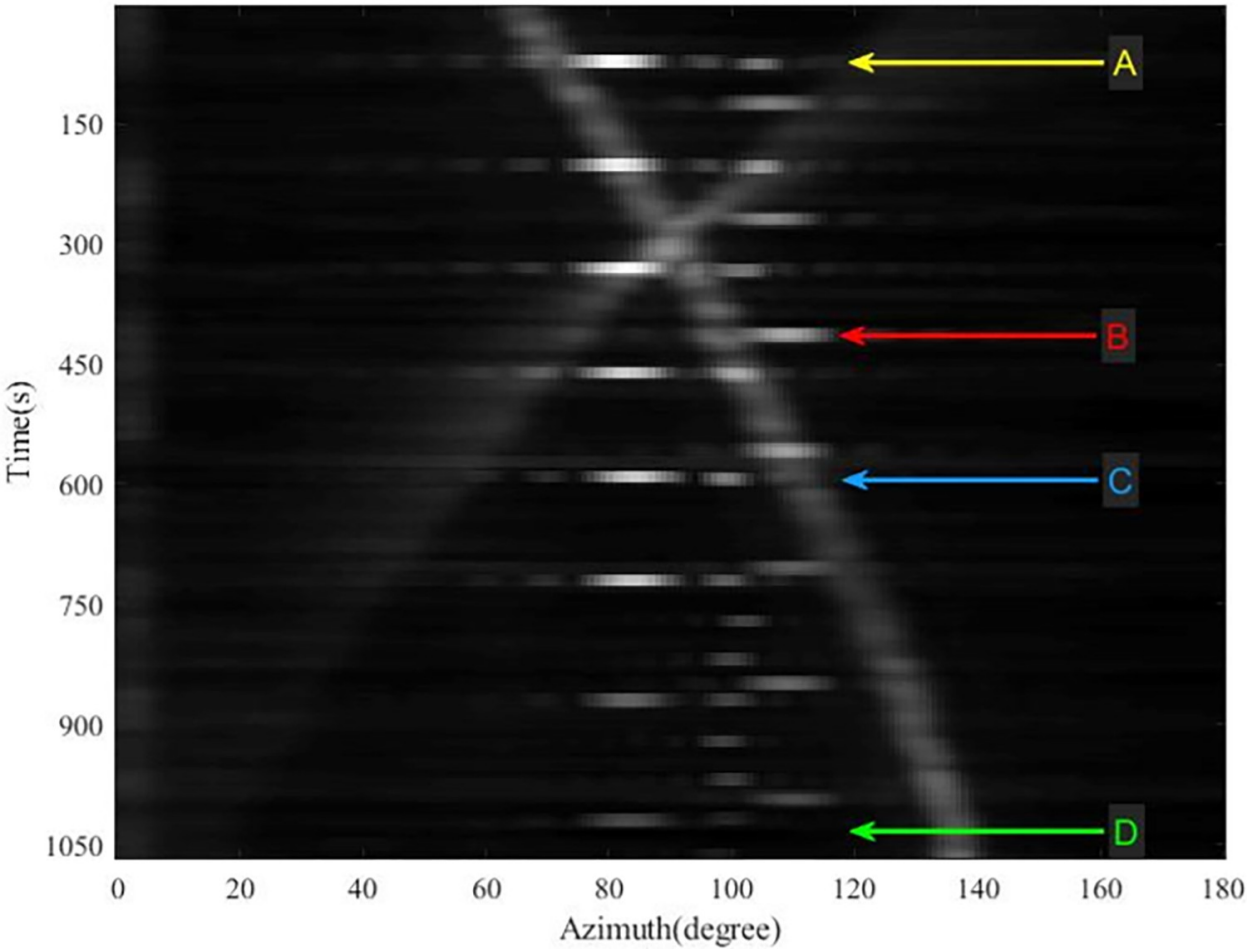
Time azimuth histogram of sea trial data obtained directly using beamforming.

Fig 13 shows the azimuth-time history chart obtained using beamforming to process the sea experiment data, from which it can be seen that multiple targets are present. The azimuth trajectory of the target is crossed, overlapped, interrupted, etc. In this paper, Multibeam Frequency Spatial Spectrogram (MFSS) is used for analysis to discriminate between different targets, and the intercepted signals are in the frequency range of 800–1600 Hz. MFSS reflects the variation of frequencies in different azimuth over a certain period of time and, in combination with the spatial spectrum, enables better identification of targets. We selected MFSS for four time-points A, B, C, and D to demonstrate the analysis process, with the aim of confirming the true azimuth-time history of the targets for subsequent comparative analysis.

**Fig 14:**
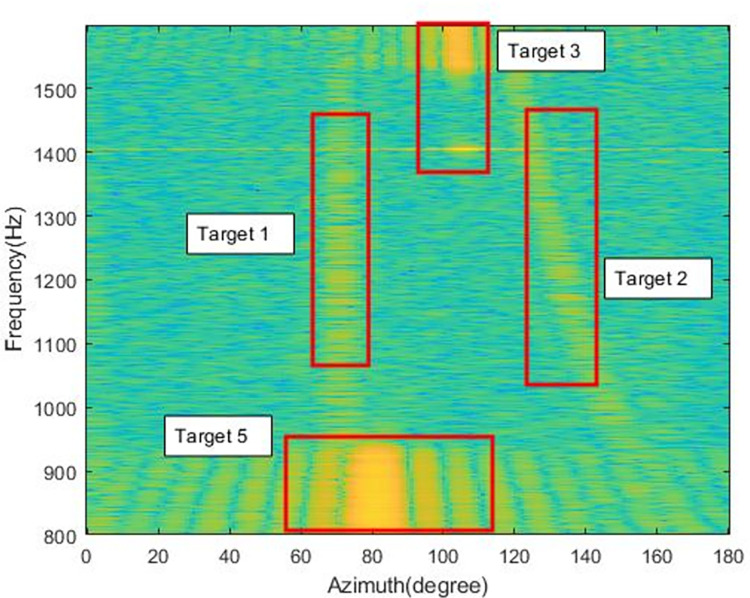
MFSS at time point A(70s).

Fig 14 shows the multi-beam LOFAR at time point A, near 70s. The x-axis in the figure is the azimuth and the y-axis is the frequency. Combining Figs 12 and 13, it can be compared to find that near 70°, there exists a target for LFM signal, tentatively designated as target 1. There is also an LFM signal target in the range of 120°-160°, which is difficult to find in the azimuth-time history chart due to energy dispersion and is tentatively designated as target 2. On the azimuth-time history chart, the stripes near 80° and 105° are not the same target, and one is at 800-900Hz and one is at 1550-1600Hz in terms of frequency. A small bright spot is formed between the two due to the interference of the target near 80°. the target near 105° is tentatively designated as target 3 and the target near 80° is tentatively designated as target 5.

**Fig 15:**
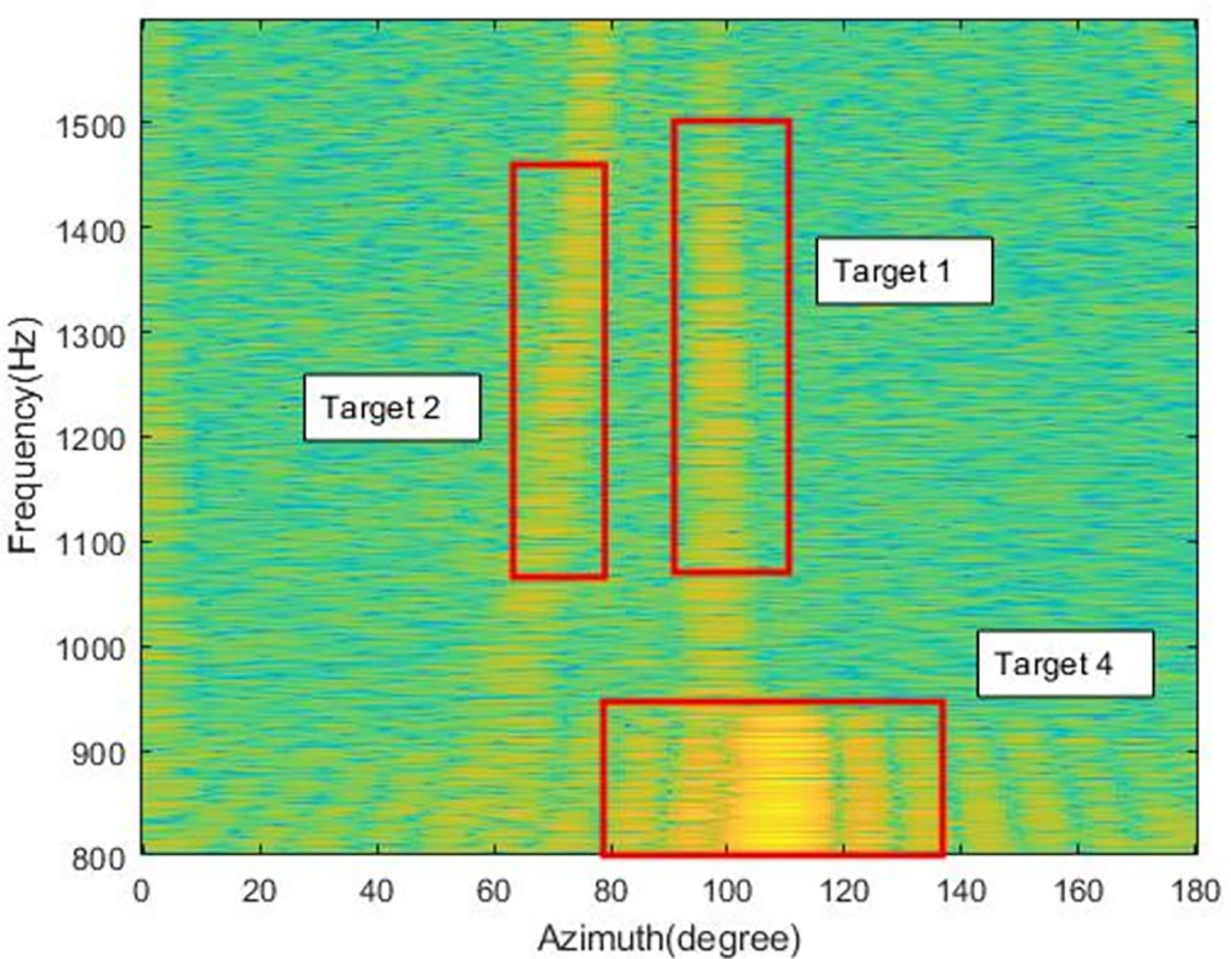
MFSS at time point B(410s).

Fig 15 shows the MFSS at time point B, near 410s. The x-axis in the figure is the azimuth and the y-axis is the frequency. From the Fig 14, it can be found that target 2 is distributed in the azimuth of 60°-80° and target 1 is present near 100°. A new target appears between 100° and 118° bearing at 800-900Hz, tentatively designated as target 4.

**Fig 16:**
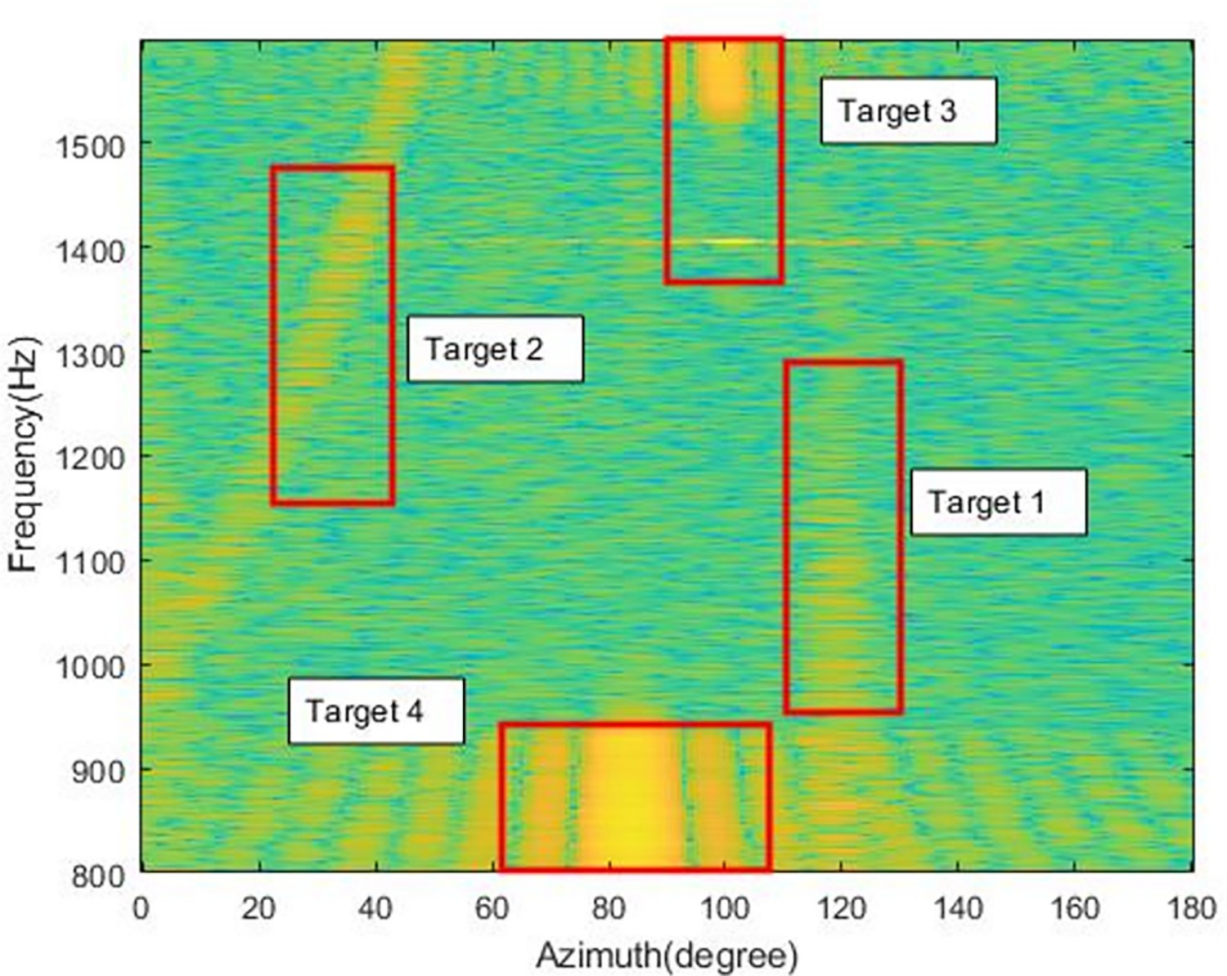
MFSS at time point C(600s).

**Fig 17:**
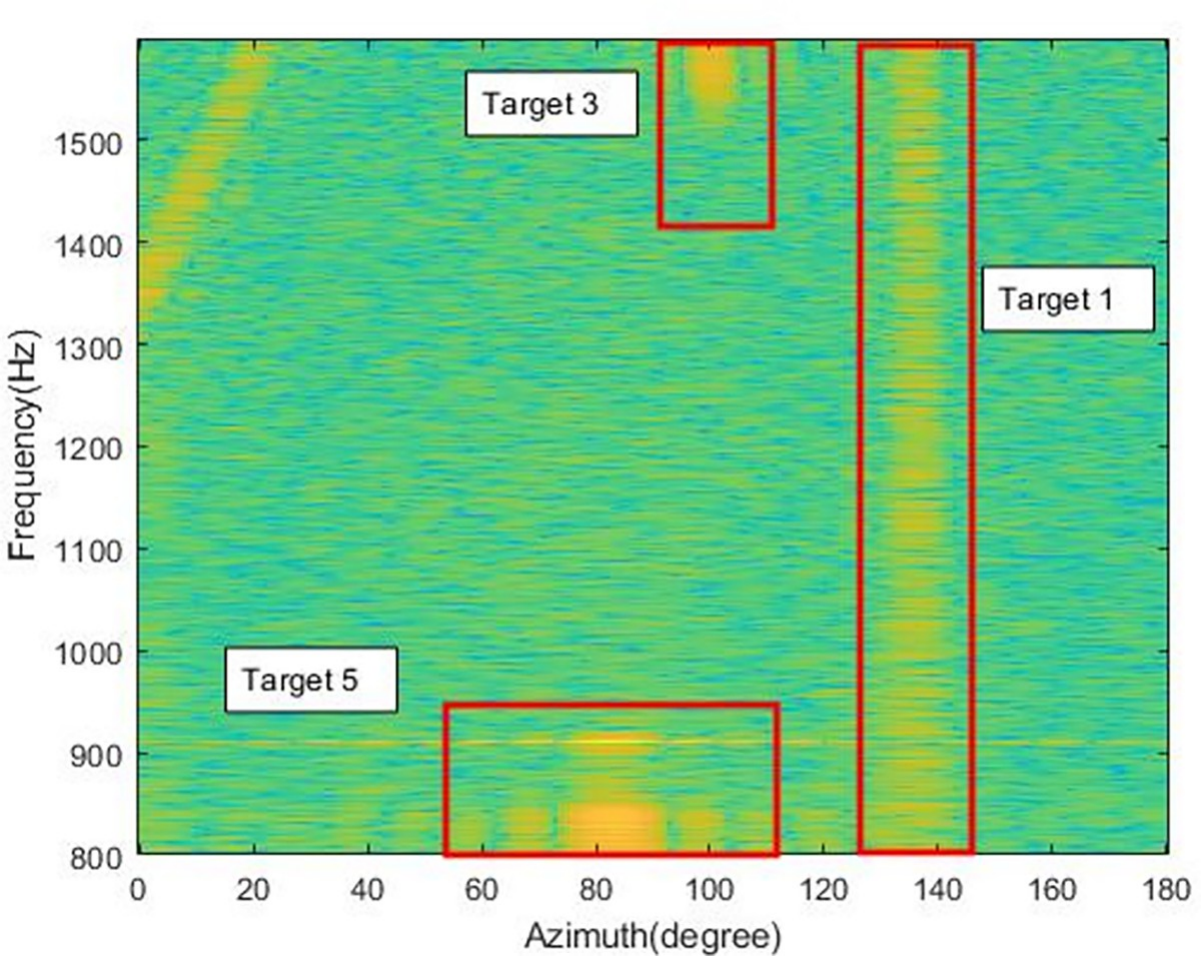
MFSS at time point D(1020s).

Figs 16 and 17 show the MFSS at time point C (600s) and time point D (1020s), respectively.

An analysis against Figs 16 and 17 allows the identification of several different targets.

Target 1: azimuth change from 70° to 140°, signal frequency range 800-1600Hz, the target beam energy is more concentrated, its azimuth trajectory is clearly discernible in Fig 12.

Target 2: azimuth change from 160° to near 20°, signal frequency range 800-1600Hz, large target azimuth span, and more dispersed target beam energy.

Target 3: azimuth change from 105° to 110°, signal frequency range 1550-1600Hz, small target azimuth span, target emergence frequency first low and then high, more concentrated beam energy.

Target 4: azimuth change from 105° to 115°, signal frequency range 800-950Hz, smaller span of target azimuth, more fixed frequency of target appearance and relatively dispersed beam energy.

Target 5: The azimuth is concentrated near 80°, the peak beam energy span is large, between 75° and 85°, and the frequency of target appearance is more fixed.

According to the previous analysis, there are five targets, among which target 1 and target 2 are continuous signals, and targets 3, 4 and 5 are impulse signals.

Among them, target 1 and target 2 can be seen from the azimuth-time history chart with obvious crossover of azimuth trajectories, marking the approximate change of azimuth of the target.

### Application of MLDE method to process sea trial data

In this paper, we select target 1 and target 2 as the main tracking objects for analysis and research, verify the tracking effect of the MLDE method, and compare it with the EKF-Max method.

**Fig 18:**
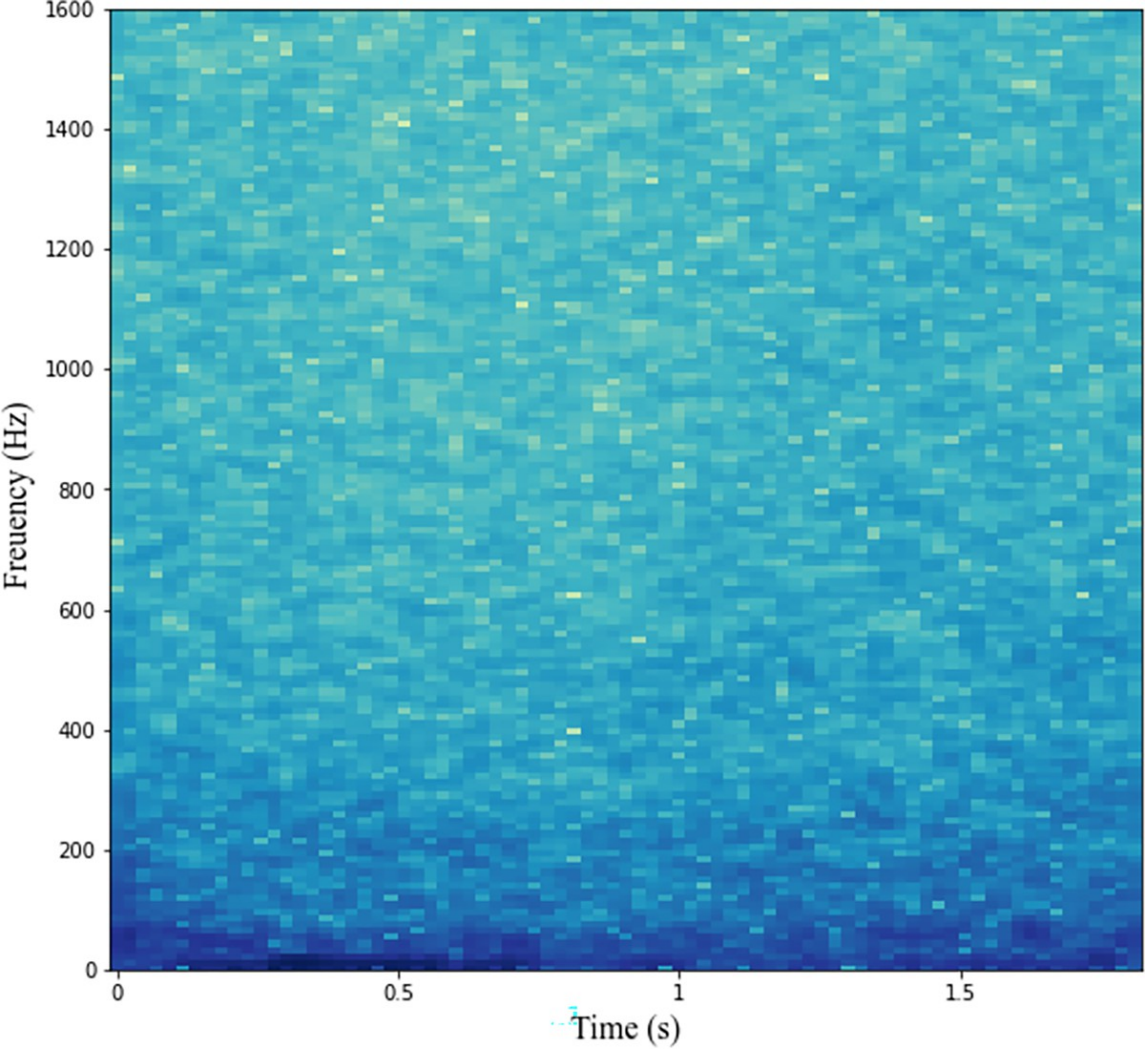
LOFAR spectrograms from sonar array raw data.

At the beginning, we directly performed LOFAR transformation on the original underwater acoustic data, and the result is shown in Fig 18. The target signal that can be analyzed in MFSS is difficult to see from the LOFAR spectrum of the original underwater acoustic data. If this raw LOFAR spectrum is used directly for identification, the effect will be poor.

**Fig 19:**
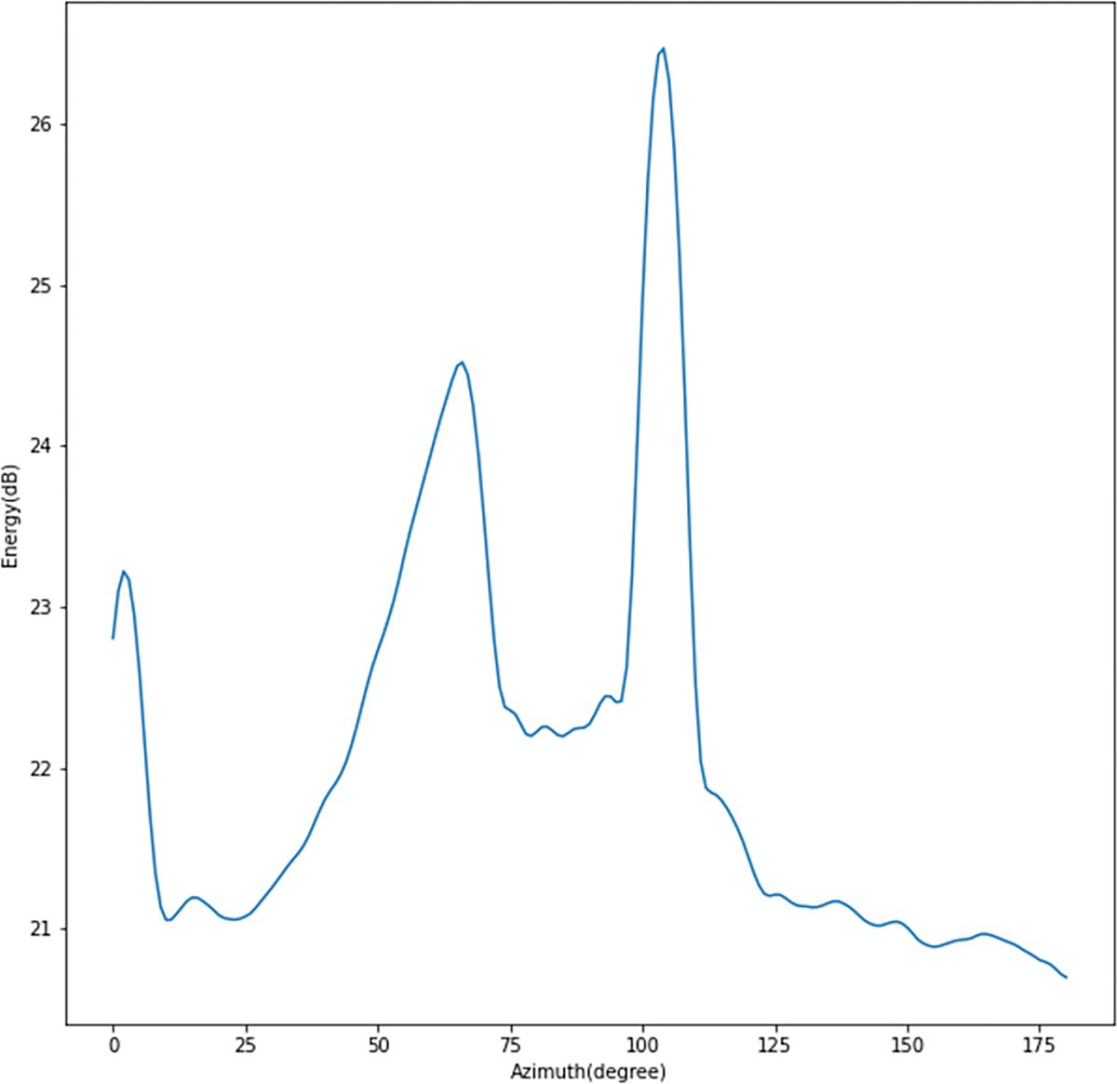
The spatial azimuth energy distribution of the original data of the sonar array at a certain moment obtained by beamforming.

We processed the sea trial data using the MLDE method proposed in this paper. First, we use beamforming to obtain a spatial azimuth energy distribution map, as shown in Fig 19. In Fig 19, the energy is strong in the azimuths of 66° and 104°, exceeding the threshold, and there are suspected targets.

**Fig 20:**
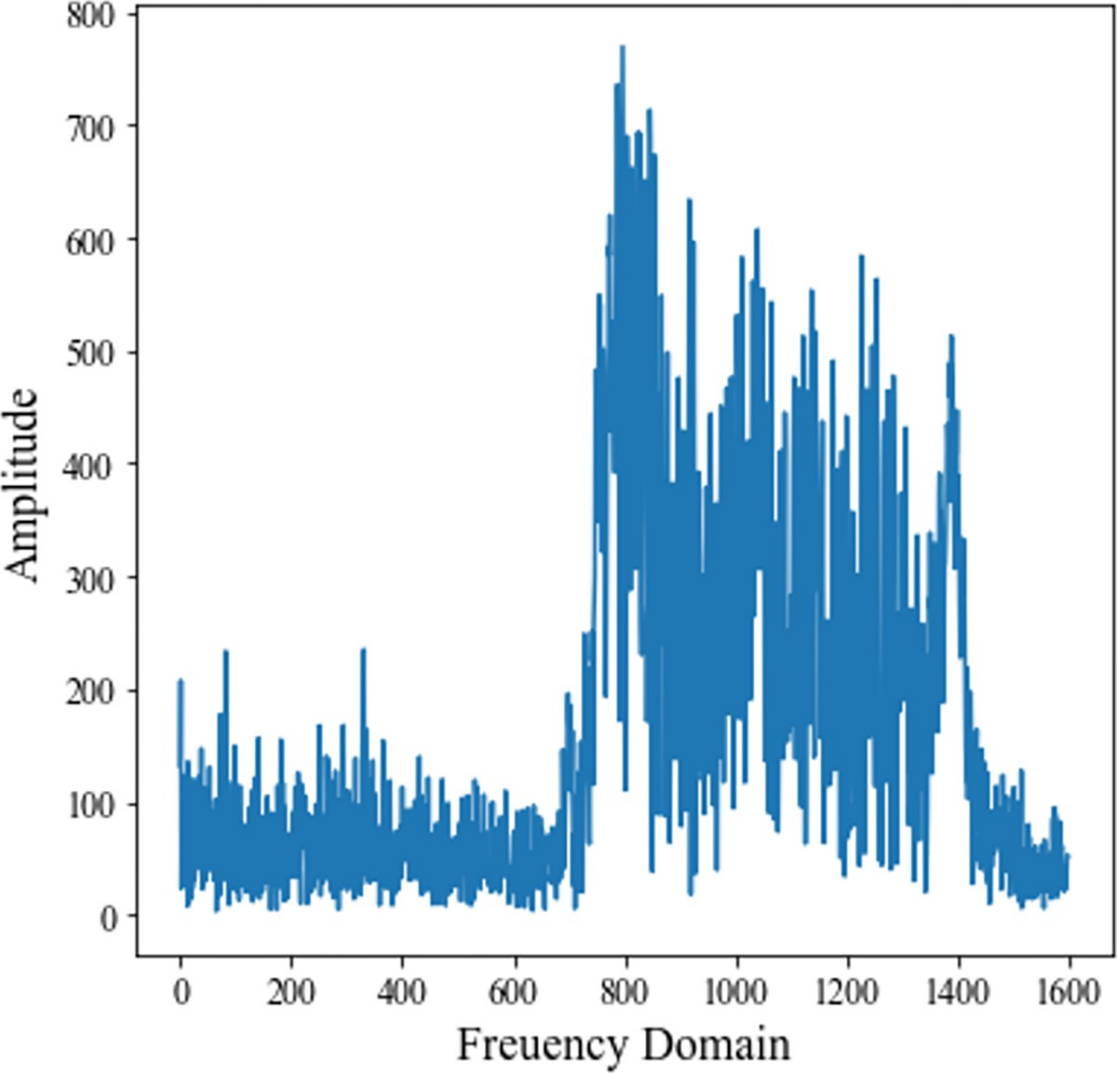
Frequency domain beam data for target.

Select a target with the azimuth of 66° to generate a multi-beam LOFAR spectrogram. First, azimuth weighting is performed on the original sonar array data to obtain the frequency-domain beam data of the target, as shown in Fig 20.

**Fig 21:**
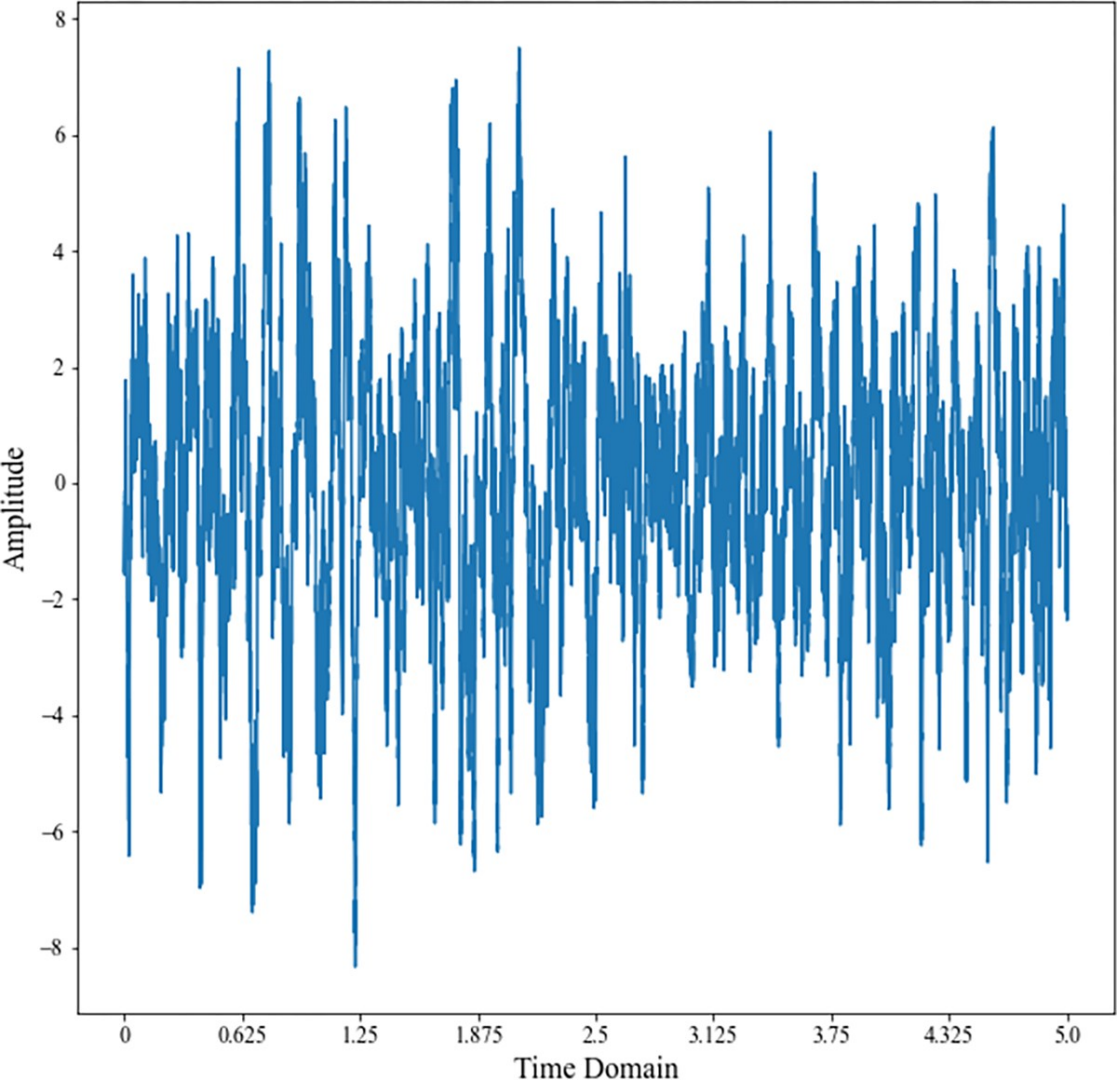
Frequency domain beam data of the target.

Convert the frequency-domain beam data to time-domain beam data and accumulate them, as shown in Fig 21.

**Fig 22:**
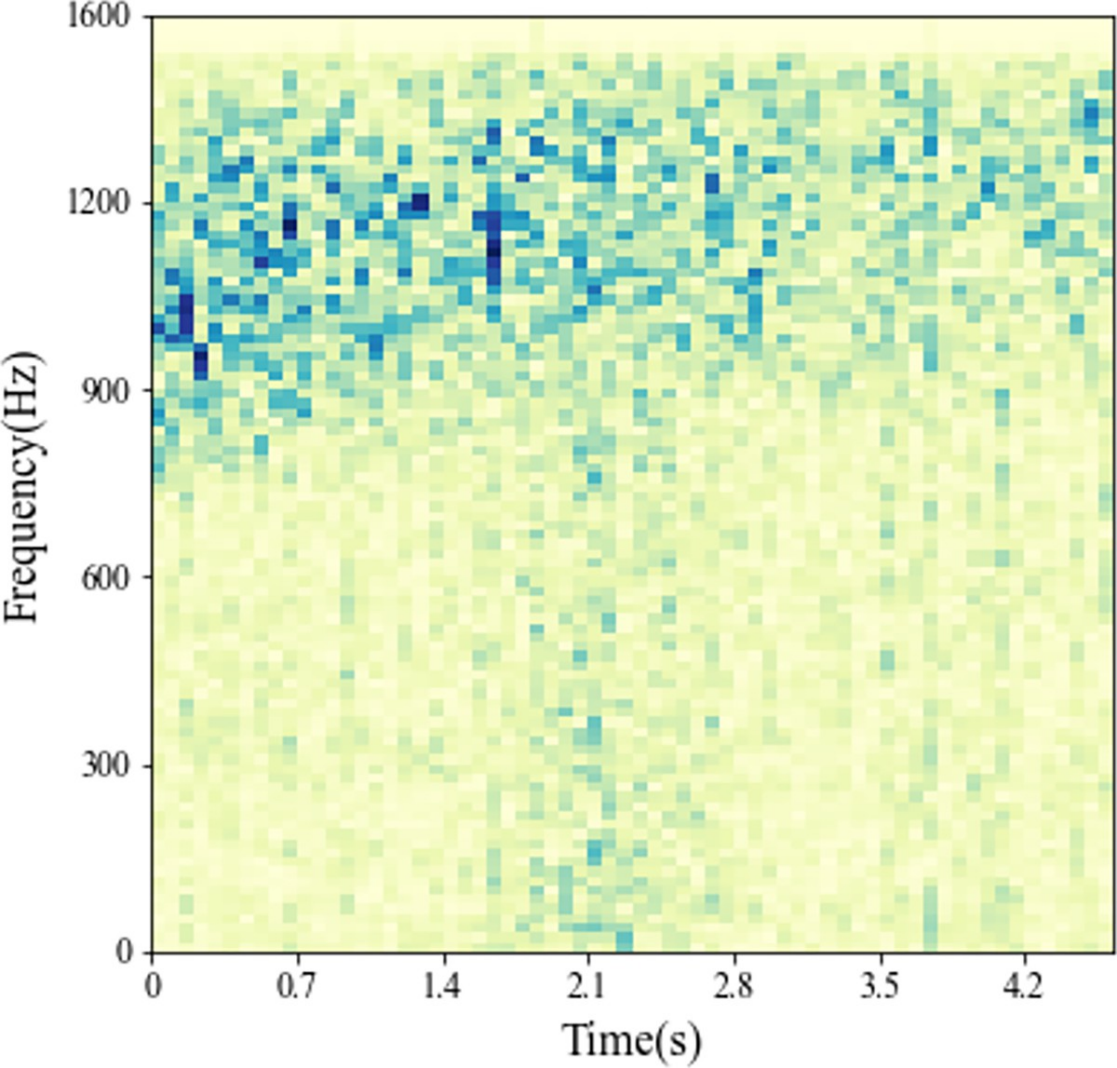
Multi-beam LOFAR spectrogram of the target.

Finally, we generate multi-beam LOFAR spectrograms from the target time-domain beam data by short-time Fourier transform. The multi-beam LOFAR spectrum is shown in Fig 22.

**Fig 23:**
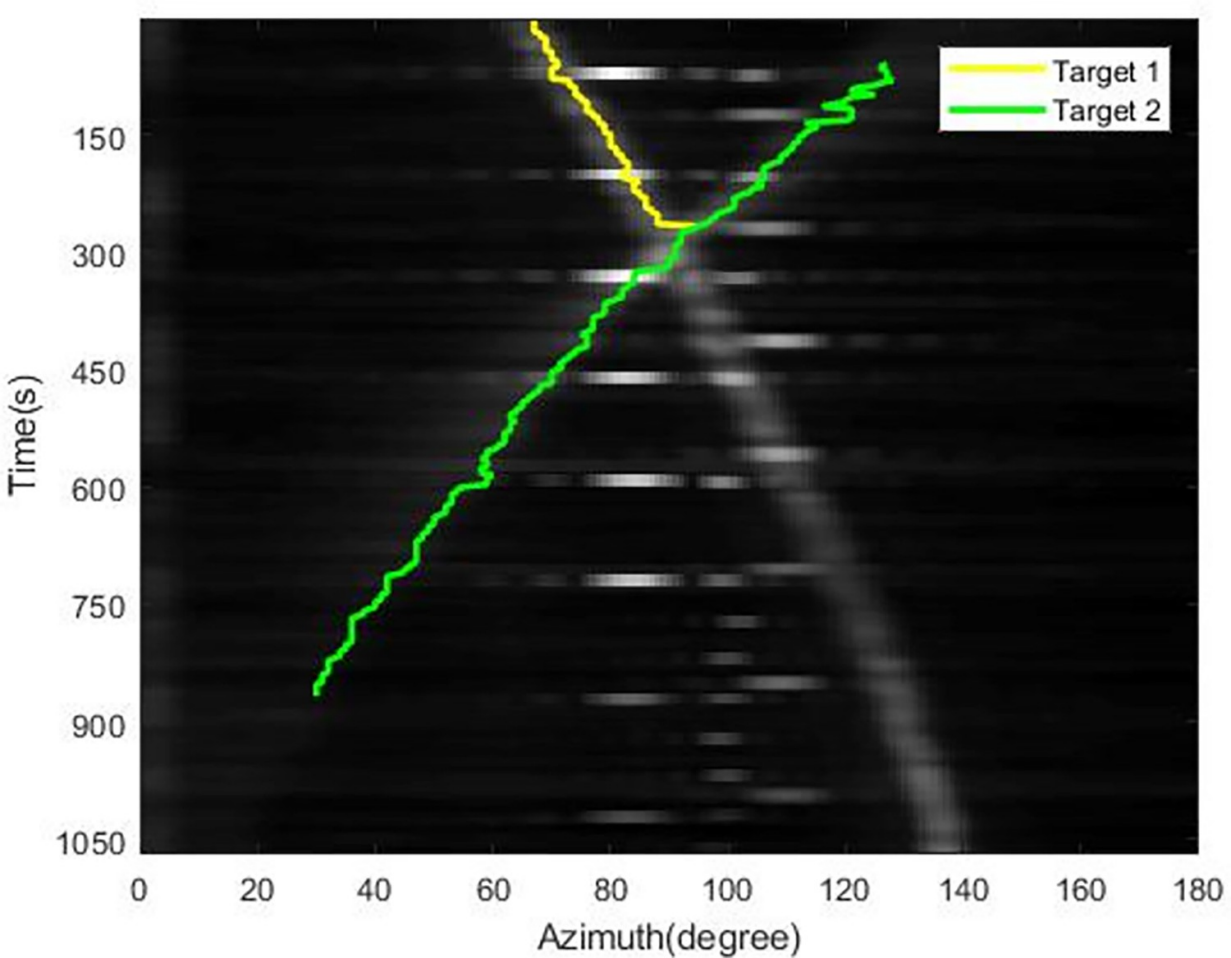
Tracking results of the EKF-Max method.

**Fig 24:**
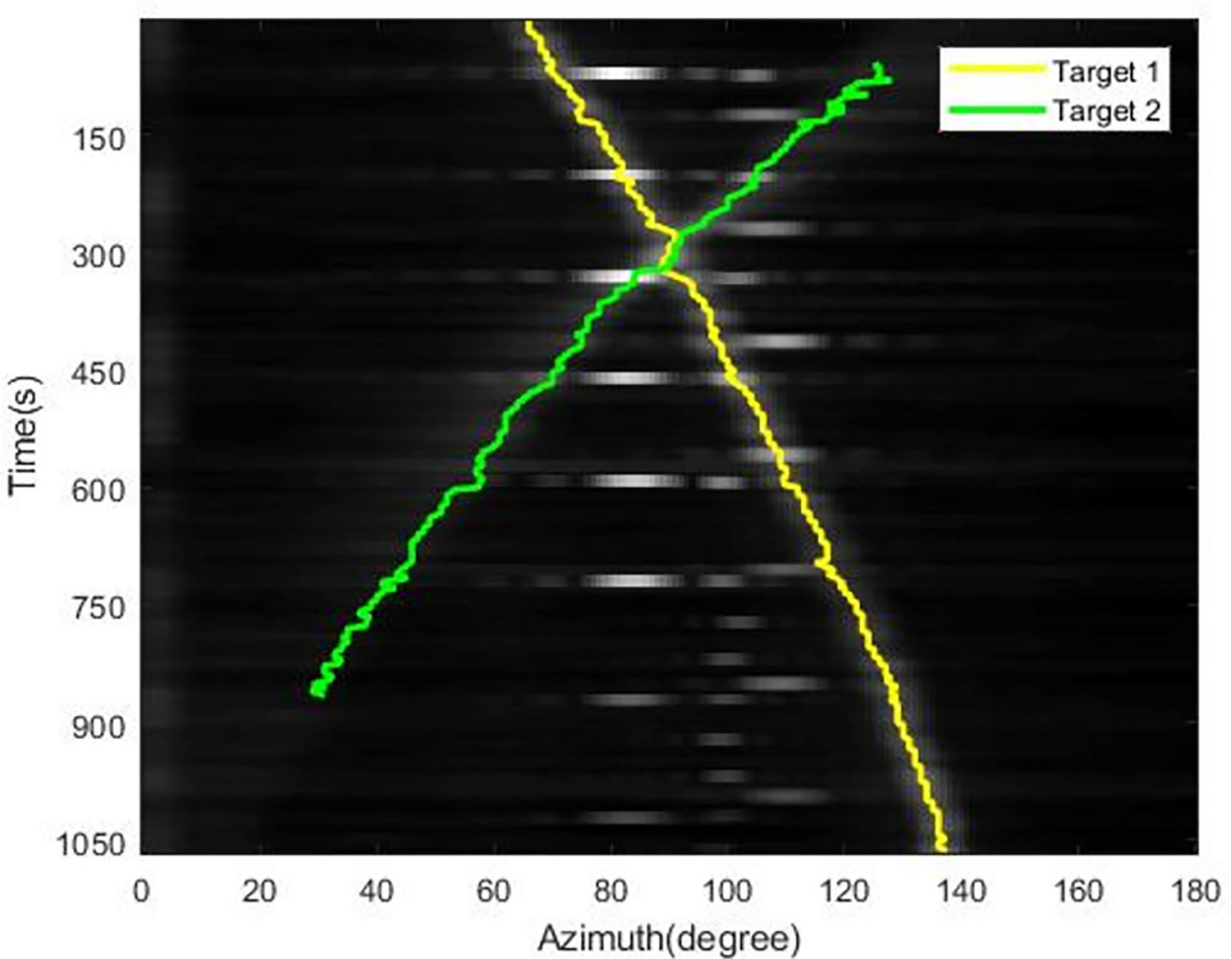
The tracking results of the MLDE method successfully identify the target where the two azimuth trajectories cross.

We trained a CNN model on the data samples, and then used the model for the recognition part of the tracking process. As a comparison, we also use the EKF-Max method to track the data, and the results are shown in Fig 23. The tracking result of MLDE method is shown in Fig 24. The tracking results of the EKF-Max method show that when the azimuths of the two targets cross, the tracker loses one of the targets. From the tracking results of the MLDE method, the target with the cross of the two azimuth trajectories can be successfully identified, and the passive tracking performance for weak targets is also improved.

## Conclusion

In this paper, the MLDE method combining multi-beam LOFAR and CNN model is proposed for passive underwater acoustic target tracking in sonar engineering. Through the processing of actual sea trial data, the MLDE method has achieved good results in tracking passive underwater acoustic targets with intersecting azimuth trajectories. The intensity of the two passive targets in the sea trial data is relatively high relative to the marine environment noise, which improves the recognition accuracy to a certain extent. Due to the limited number of sea trial data samples, more complex and richer actual data verification is lacking.

In the future, there are still some problems that need to be solved in the real-time passive tracking of underwater acoustic targets. At present, the energy threshold and continuous line spectrum are mainly used in judging the target to distinguish the target from the noise. How to automatically judge the target and confirm that the target is worth tracking is a problem worthy of research. Secondly, when the self-radiation noise of the underwater acoustic target is in the low frequency range or covered by the environmental noise, how to distinguish the target is also a problem for future practical applications. In the actual sea trial data analysis part of this paper, we use the MFSS spectrum to manually confirm the target, and the accuracy rate is relatively high. However, because the frequency distribution of multiple targets exists on the MFSS spectrum at the same time, and at different times, due to the movement of the target, the shape of the target on the MFSS spectrum will change at any time. This is also a direction for using deep learning to solve passive tracking of underwater acoustic targets in the future.
